# Chrysophanol-8-O-glucoside protects mice against acute liver injury by inhibiting autophagy in hepatic stellate cells and inflammatory response in liver-resident macrophages

**DOI:** 10.3389/fphar.2022.951521

**Published:** 2022-09-06

**Authors:** Tao Wang, Zhuo Lu, Xin-Hui Qu, Zi-Ying Xiong, Ya-Ting Wu, Yong Luo, Zi-Yu Zhang, Xiao-Jian Han, Cai-Feng Xie

**Affiliations:** ^1^ Institute of Geriatrics, Jiangxi Provincial People’s Hospital, The First Affiliated Hospital of Nanchang Medical College, Nanchang, China; ^2^ School of Basic Medical Sciences, Nanchang University, Nanchang, China; ^3^ Department of Thoracic Surgery, The First Affiliated Hospital of Nanchang University, Nanchang, China; ^4^ Department of Neurology, Jiangxi Provincial People’s Hospital, The First Affiliated Hospital of Nanchang Medical College, Nanchang, China; ^5^ Department of Pathology, Jiangxi Maternal and Child Health Hospital, Nanchang, China; ^6^ Key Laboratory of Women’s Reproductive Health of Jiangxi, Jiangxi Maternal and Child Health Hospital, Nanchang, China; ^7^ Department of Pharmacology, School of Pharmaceutical Science, Nanchang University, Nanchang, China

**Keywords:** acute liver injury, lipopolysaccharide, chrysophanol-8-O-glucoside, autophagy, oxidative stress

## Abstract

Acute liver failure (ALF) is an unfavorable condition characterized by the rapid loss of liver function and high mortality. Chrysophanol-8-O-glucoside (CPOG) is an anthraquinone derivative isolated from *rhubarb*. This study aims to evaluate the protective effect of CPOG on lipopolysaccharide (LPS)/D-GalN-induced ALF and its underlying mechanisms. LPS/D-GalN-induced mice ALF model and LPS treatment model in RAW 264.7 and LX2 cells were established. It was found that CPOG ameliorated LPS/D-GalN-induced liver injury and improved mortality as indicated by Hematoxylin-eosin (H&E) staining. Molecularly, qPCR and ELISA results showed that CPOG alleviated LPS/D-GalN-induced release of alanine aminotransferase and aspartate transaminase and the secretion of TNF-α and IL-1β *in vivo*. LPS/D-GalN-induced intracellular ROS production was also attenuated by CPOG in liver tissue. Further, CPOG attenuated ROS generation and inhibited the expression of p-IκB and p-p65 as well as the expression of TNF-α and IL-1β stimulated by LPS in RAW 264.7 cells. In addition, CPOG alleviated LPS-induced up-regulation of LC3B, p62, ATG5 and Beclin1 by attenuating ROS production and inhibiting MAPK signaling in LX2 cells. Taken together, our data indicated that the CPOG protected against LPS/D-GalN-induced ALF by inhibiting oxidative stress, inflammation response and autophagy. These findings suggest that CPOG could be potential drug for the treatment of ALF in clinic.

## 1 Introduction

The liver is generally comprised of non-parenchymal cells (NPC) and parenchymal cells (hepatocytes). Non-parenchymal cells include populations of Kupffer cells, hepatic stellate cells (HSCs), liver sinusoidal endothelial cells (LSEC) and intrahepatic lymphocytes (Racanelli and Rehermann, 2006). The crosstalk between hepatocytes and non-parenchymal cells is key to liver homeostasis, while it is commonly believed that non-parenchymal cells can be primary targets for hepatotoxins and mediate physiological response to endocrine and immune signal (Robinson et al., 2016). Acute liver failure (ALF) is characterized by loss of liver function that occurs rapidly in a person who has no pre-existing liver disease (Krawitz et al., 2018). Apart from liver transplantation, there is no effective remedy (Patel et al., 2018). Therefore, the need for efficient drugs for treating acute liver failure is urgent.

Lipopolysaccharides (LPS), also known as endotoxins, could result in systemic inflammatory response syndrome and multiple organ failure (Jirillo et al., 2002). A well-established mice model of macrophage-mediated ALF is gavage administration of LPS and D-galactosamine (GalN) (Nakama et al., 2001; Wan et al., 2008). LPS quickly stimulated reactive oxygen species (ROS) and impart damage to the both parenchymal and non-parenchymal cells (Hsu and Wen, 2002). LPS can also stimulate inflammatory cells especially macrophages to release various inflammatory mediators like TNF-α and IL-1β by activating NF-κB signaling pathway, which further escalated the liver damage by feedback mechanism (Muniandy et al., 2018). Autophagic cell deaths mediated by oxidative stress also play important role in the LPS-induced pathogenesis (Yuan et al., 2009; Li et al., 2015).

Chrysophanol is a free anthraquinone compound isolated from *Rheum genus*. Recent studies showed that Chrysophanol may exert anti-cancer effects (Lu et al., 2010; Ni et al., 2014), anti-inflammation activity (Kim et al., 2010) and offer neuroprotection (Chu et al., 2018). Previous study showed that chrysophanol has protective effect on LPS-induced ALF, though the precise mechanism is not clear (Jiang et al., 2016). However, free chrysophanol has potential hepatoxicity and nephrotoxicity (Yu et al., 2011; Wang et al., 2017). Animals administered of chrysophanol had adverse reactions such as bowel sounds, nausea, vomiting, abdominal pain and diarrhea. Thus, structural modifications of chrysophanol have been utilized to improve therapeutic efficacy and alleviate its side effect (Shrestha et al., 2014; Mondal et al., 2015).

Chrysophanol-8-O-glucoside (CPOG) is glycosylated chrysophanol, whose content is higher than chrysophanol in *Rheum genus* (Wang et al., 2013). *In vitro* study showed that CPOG protected against hepatic fibrosis through STAT3 signaling (Park et al., 2020). However, pharmacology activity *in vivo* of CPOG remains largely unknown.

In this study, we aim to investigate the protective effect of CPOG against acute liver failure and to explore the underlying mechanism with respect to oxidative stress, inflammation response and autophagy. It was found that CPOG ameliorated LPS/D-GalN-induced liver damage and improved survival rate. CPOG alleviated LPS-induced oxidative stress both *in vivo* and *in vitro*. Moreover, CPOG showed anti-oxidant effects and inhibited the release of inflammatory cytokines by the inactivation of NF-κB signaling pathway in RAW264.7 cells. In addition, CPOG blocked LPS-induced activation of MAPK signaling, therefore attenuated the expression of autophagy-related proteins and LC3 puncta formation in LX2 cells. In conclusion, our study indicate that CPOG could protect against ALF by inhibiting oxidative stress, inflammation response and autophagy. We propose that CPOG could be a potential drug for ALF treatment in clinic.

## 2 Materials and methods

### 2.1 Reagents

Chrysophanol-8-O-glucoside (PHL84206), N-Acetyl-L-cysteine (A9165), LPS (L4391) and D-GalN (G1639) were purchased from Sigma. Primary antibodies against ATG5 (10181-2-AP), Beclin1 (11306-1-AP), β-actin (66,009-1-lg), LC3B (18725-1-AP), and ERK (16443-1-AP) were purchased from Proteintech. Antibody against p-ERK (sc-101761) was purchased from Santa Cruz. Antibody against p62 was purchased from OriGene (TA502127). NF-κB Pathway Sampler Kit (#9936) was purchased from Cell signaling technology.

### 2.2 Animals

BALB/c mice (6–8 weeks age) were purchased from Hunan SJA Laboratory Animal Co., Ltd. (Changsha, Hunan Province, China), and fed in the Specific Pathogen Free animal facility in the Institute of Life Science at Nanchang University in strict accordance with the recommendations in the Guide for the Care and Use of Laboratory Animals of the Nanchang University in China (IACUC approval No. SYXK 2015-0001).

### 2.3 LPS-D/GalN-induced acute liver injury

BALB/c mice injected with D-GalN (750 mg/kg) and LPS (0.35 mg/kg, *Salmonella abortus equi*) were treated with CPOG or equal volume of vehicle as previously reported (Nakama et al., 2001). Blood plasma was collected 8 h after administration under isoflurane anesthesia. ELISA assay was used to analyze the levels of serum TNF-α and IL-1β according to instruction manual (R&D Systems, Minneapolis, Minnesota, United States).

For survival rate calculation, NAC (100 mg/kg), CPOG (20 or 40 mg/kg) were respectively pre-administrated 1 h before LPS/D-GalN injection. Then mice were injected with lethal dose of D-GalN (750 mg/kg) and LPS (1.5 mg/kg) (Tiegs et al., 1994). Every 2 h for 24 h, the number of dead mice was counted.

### 2.4 Cell culture

Mouse macrophage-like cell line (RAW264.7) were obtained from the Type Culture Collection of the Chinese Academy of Sciences (Shanghai, China). Cells were cultured in DMEM (Gibco) supplemented with 10% heat inactivated FBS (Excel, FCS100) and cultured at 37°C with 5% CO_2_. Hepatic cell lines (LO2 and LX2) were cultured in DMEM (Gibco) supplemented with 10% FBS (Excel, FCS100) at 37°C in the presence of 5% CO_2_.

### 2.5 Histological analysis

Under pentobarbital anesthesia (100 mg/kg, intraperitoneal injection), livers were dissected (*n* = 8 per group) from animals. Then, the tissues were fixed and subjected to immunohistochemical staining and Hematoxylin-eosin (H&E) staining conducted by Wuhan Servicebio Technology Co., Ltd. According to Heijnen’s technique, extent of liver injury was evaluated (Johnson et al., 1992; Heijnen et al., 2003).

### 2.6 Immunofluorescence and immunohistochemistry

Mice livers were removed from animals under anesthetic, fixed in 4% paraformaldehyde, and embedded in paraffin. For immunofluorescence assay, paraffin sections were dewaxed in xylene, and rehydrated in ethanol. Then, the sections were washed with PBS, and incubated in boiling antigen retrieval solution for 15 min. The sections were blocked with 1% BSA and 5% serum in PBS at room temperature for 1 h, and then incubated with indicated primary antibody at 4°C overnight. After washed with PBS for 3 times, the sections were incubated with secondary antibody at room temperature for 1 h. The sections were washed and counterstained with DAPI. The images were analyzed by ImageScope software.

For immunohistochemistry, the sections were dewaxed, rehydrated and treated the same as immunofluorescence assay till the incubation with the second antibody. The sections were then incubated with biotinylated secondary antibody for 1 h at room temperature. Then, the slides were washed with PBS and incubated with ABC Reagent (Vectorlabs) for 30 min. After washed in PBS for 3 times, the sections were incubated with fresh prepared DAB substrate solution. The sections were counterstained with hematoxylin solution, and the images were analyzed by ImageScope software.

### 2.7 Western blot analysis

For western blot assay, proteins were separated on gel where appropriate. And then transferred to PVDF membranes (Millipore, IPVH00010), which were blocked with 5% BSA (Genview, FA016). Next, PVDF membranes were incubated with the indicated antibodies. Lastly, PVDF membranes were incubated with anti-rabbit (Thermo Fisher Scientific, 31460) or anti-mouse (Thermo Fisher Scientific, 31430) secondary antibodies. Western blot results were obtained by digital gel image analysis system (TANON 5500) and Pro-Light chemiluminescence detection kit (TIANGEN, PA112-01).

### 2.8 Quantitative RT-PCR

TRIzol reagent (Invitrogen) were used to extract total RNA of the cells. Then, production of the cDNA was used by the PrimeScript RT reagent kit with gDNA eraser (Takara) according to the instruction manual. The sequences of the probes used to quantify TNF-α (Genbank No. NM_013693.3) mRNA levels were: 5’ -CTC​CAG​GCG​GTG​CCT​ATG​TCT-3’ (sense); 5’ -CTC​CTC​CAC​TTG​GTG​GTT​TGC-3’ (antisense). The sequences of the probes used to quantify IL-1β (Genbank No. NM_008361.4) mRNA levels were: 5’ -GTG​TCT​TTC​CCG​TGG​ACC​TTC-3’ (sense); 5’ -TCA​TCT​CGG​AGC​CTG​TAG​TGC-3’ (antisense). Quantitative RT-PCR was performed using SYBR Green dye and the expression of GAPDH was used as control.

### 2.9 Measurement of intracellular ROS level

ROS levels were measured according to the protocol from the CM-H2DCFDA Cellular ROS Detection Assay Kit (Invitrogen). Cells or fresh liver tissue were homogenized with NP40 lysis buffer at 4°C for 30 min and centrifuged at 10,000 g for 20 min. The supernatants were incubated in the presence of 10 μM CM-H2DCFDA at 37°C for 15 min. Fluorescence intensity was measured at an excitation wavelength of 485 nm and an emission wavelength of 535 nm. Supernatant (5 μL) was used for protein quantification using the Bradford assay. We normalized the fluorescence intensity by dividing the total protein. The total protein was equal to the volume of the supernatant × the protein concentration.

### 2.10 Statistical analysis

The data were showed as mean ± S.D. The results obtained from triplicate-independent experiments. Significance was determined by one-way ANOVA or a two-tailed unpaired Student’s *t* test where appropriate. *p* < 0.05 were considered significant.

## 3 Results

### 3.1 CPOG protected mice from LPS/D-GalN induced ALF

**FIGURE 1:**
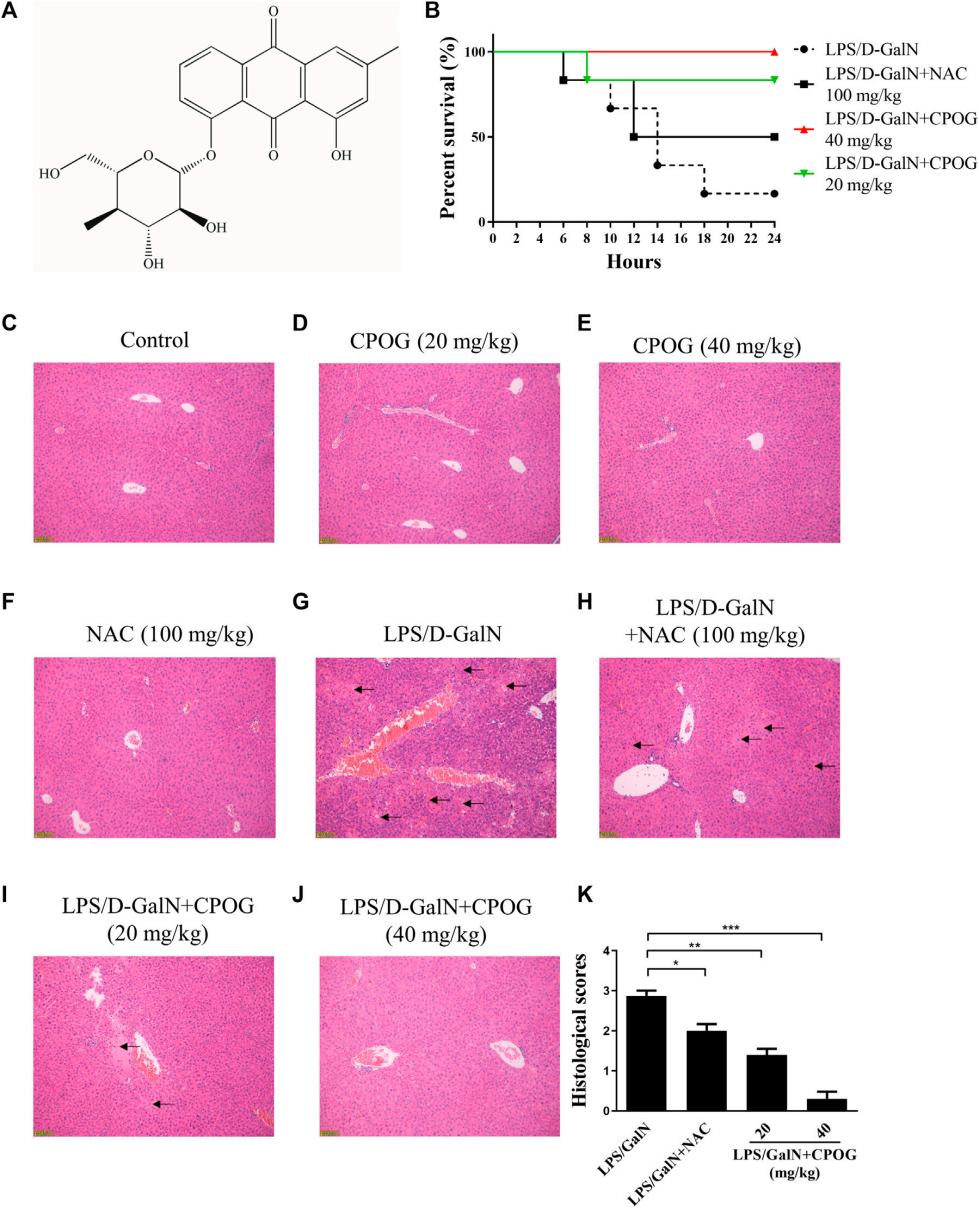
CPOG ameliorates LPS/D-GalN-induced ALF. **(A)** Chemical structure of CPOG. **(B)** Kaplan-Meier method was used to create the survival curves after LPS/D-GalN injection. **(C–G)** Liver sections were subjected to H&E staining. Representative photographs were shown from vehicle mice **(C)**, mice treated with CPOG (20 mg/kg) **(D)**, mice treated with CPOG (40 mg/kg) **(E)**, mice treated with NAC **(F)**, mice treated with LPS/D-GalN **(G)**, mice treated with LPS/D-GalN and NAC **(H)**, mice treated with LPS/D-GalN and CPOG (20 mg/kg) **(I)**, mice treated with LPS/D-GalN and CPOG (40 mg/kg) **(J)**. Arrows represent pathological changes in liver tissue. **(K)** Histological scores of liver sections were determined. Values represent mean ± S.D. one-way ANOVA test was used to determine the significances. *p <0.05, **p < 0.01, ***p < 0.001.

Chemical structure of CPOG was shown in Figure 1A. To evaluate the effects of CPOG on LPS/D-GalN-induced lethality, mice were gavage administered with LPS and GalN as previously reported (Nakama et al., 2001). As was shown in Figure 1B, the mortality of the LPS/D-GalN treated group was 83.4%. 20 or 40 mg kg^−1^ CPOG administration significantly decreased the mortality (16.7% and 0%), which was better than that treated with 100 mg/kg N-acetyl-L-cysteine (NAC) (50%). NAC is a thiol antioxidant and employed as positive control (Wang et al., 2007). Mice administered with CPOG or NAC alone showed no mortality (Supplementary Figure S1).

Then, H&E staining was performed to detect the morphology changes in liver tissue. Normal mice or mice administered with CPOG or NAC alone showed no pathological changes in liver tissue (Figures 1C–F). However, massive immigration of inflammatory cells into sinusoids, destruction of hepatic architecture, hepatocyte necrosis and congestion were observed at 8 h after LPS/D-GalN treatment (Figure 1G). Mice co-treated with LPS/D-GalN and NAC or CPOG showed a slight inflammatory cells immigration and mild hepatocytes necrosis (Figures 1H–J). Furthermore, compared with the LPS/D-GalN treated mice, the histological scores of mice treated with CPOG decreased significantly (Figure 1K). These results indicate that CPOG protected against LPS/D-GalN-induced ALF.

### 3.2 CPOG inhibited aminotransferases and proinflammatory cytokines production in LPS/D-GalN induced ALF model

**FIGURE 2:**
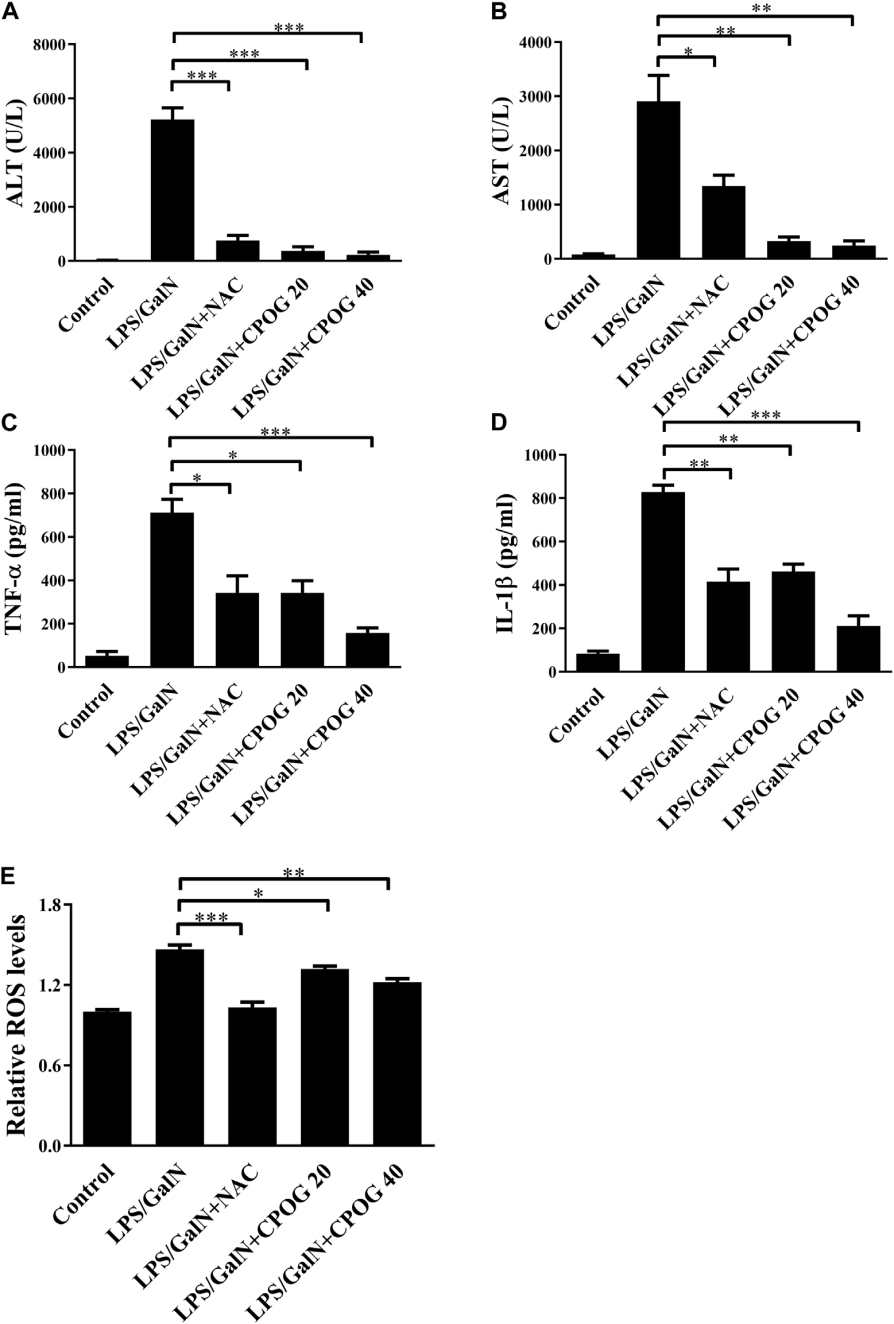
CPOG attenuated inflammatory response in LPS/D-GalN-induced ALF model. **(A,B)** 8 h after LPS/D-GalN injection, the serum levels of ALT and AST were detected. **(C,D)** Serum levels of TNF-α and IL-1βwere detected by ELISA at 8 h after LPS/D-GalN injection. **(E)** ROS levels in liver tissue were determined by CM-H2DCFDA at 8 h after LPS/D-GalN injection. Values represent mean ± S.D. Significance was determined by one-way ANOVA test. Data are representative of three independent experiments. *p < 0.05, **p < 0.01, ***p < 0.001.

Alanine aminotransferase (ALT) and aspartate transaminase (AST) are key indicators of liver function (Kim et al., 2020). Our results showed an increased levels of ALT and AST in LPS/D-GalN treated group (5226.9 U/L and 2903.6 U/L), which were 26.8 U/L and 80.6 U/L in control group, indicating that mice administered with LPS/D-GalN had developed serious hepatocytes necrosis. By contrast, the ALT levels in 20 or 40 mg kg^−1^ of CPOG-treated group decreased to 376.2 U/L (*p* < 0.001) or 223.0 U/L (*p* < 0.001). The AST levels in 20 or 40 mg/kg of CPOG -treated group decreased to 326.8 U/L (*p* < 0.001) or 247.0 U/L (*p* < 0.01) (Figures 2A,B). These results indicated that CPOG administration could ameliorate the increase of ALT and AST induced by D-GalN/LPS. IL-1β and TNF-α are the most important proinflammatory cytokines that promote the secretion of downstream proinflammatory mediators (Shen et al., 2020). The serum levels of IL-1β and TNF-α were also increased after LPS/D-GalN injection. However, co-treatment with CPOG or NAC inhibited the release of TNF-α and IL-1β compared with that in LPS/D-GalN treated group (Figures 2C,D). Furthermore, elevated intracellular ROS level plays an important role in the pathogenesis of LPS-induced ALF (Jiang et al., 2018). CM-H2DCFDA staining results showed that LPS/D-GalN quickly stimulated ROS in liver tissue, which could be attenuated by NAC treatment. Co-treatment with CPOG also inhibited LPS/D-GalN-induced ROS production (Figure 2E), indicating that CPOG antagonized LPS/D-GalN-induced ALF by alleviating ROS generation.

### 3.3 CPOG inhibited autophagy in LPS/D-GalN-induced ALF model

**FIGURE 3:**
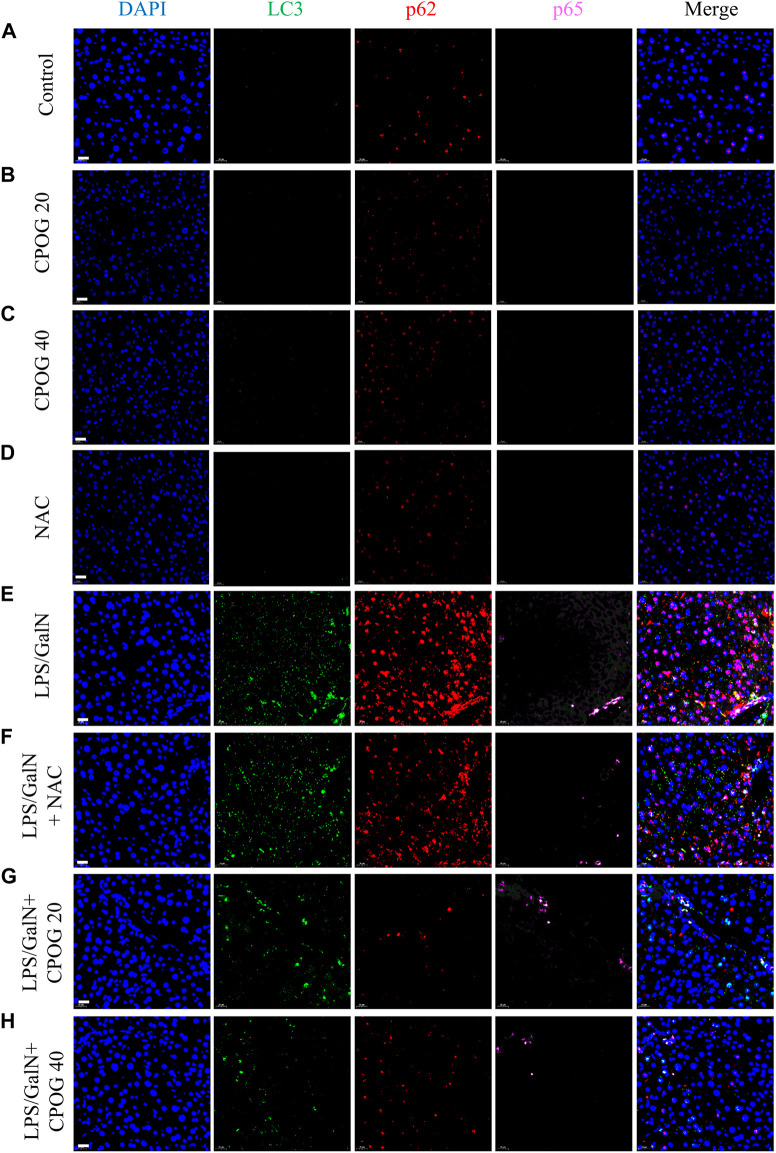
CPOG inhibited the expression of LC3B and p62 in LPS/D-GalN-induced ALF model. **(A–H)** Immunofluorescence imaging was performed with the indicated antibodies. The nucleus was stained with DAPI (blue color). Representative photographs were shown from vehicle mice **(A)**, mice treated with CPOG (20 mg/kg) **(B)**, mice treated with CPOG (40 mg/kg) **(C)**, mice treated with NAC **(D)**, mice treated with LPS/D-GalN **(E)**, mice treated with LPS/D-GalN and NAC **(F)**, mice treated with LPS/D-GalN and CPOG (20 mg/kg) **(G)**, mice treated with LPS/D-GalN and CPOG (40 mg/kg) **(H)**. Scale bar = 20 µm.

It was reported that excessive autophagic response contributed to LPS-induced liver injury (Kong et al., 2017). Therefore, immunofluorescence staining was applied to detect the expression of autophagy-related proteins in liver tissue sections. Both the expression of LC3B and p62 were increased after LPS/D-GalN injection, as compared with normal mice and mice treated with NAC or CPOG alone (Figures 3A–E), indicating an increased autophagic activity. The co-treatment with LPS/D-GalN and NAC or CPOG (20 or 40 mg/kg) decreased expression of LC3B and p62 (Figures 3F–H). p65 is an important executor of NF-κB signaling that regulates the expression of pro-inflammatory cytokines (Li et al., 2021). However, the expression of p65 showed no significant change following the LPS administration.

**FIGURE 4:**
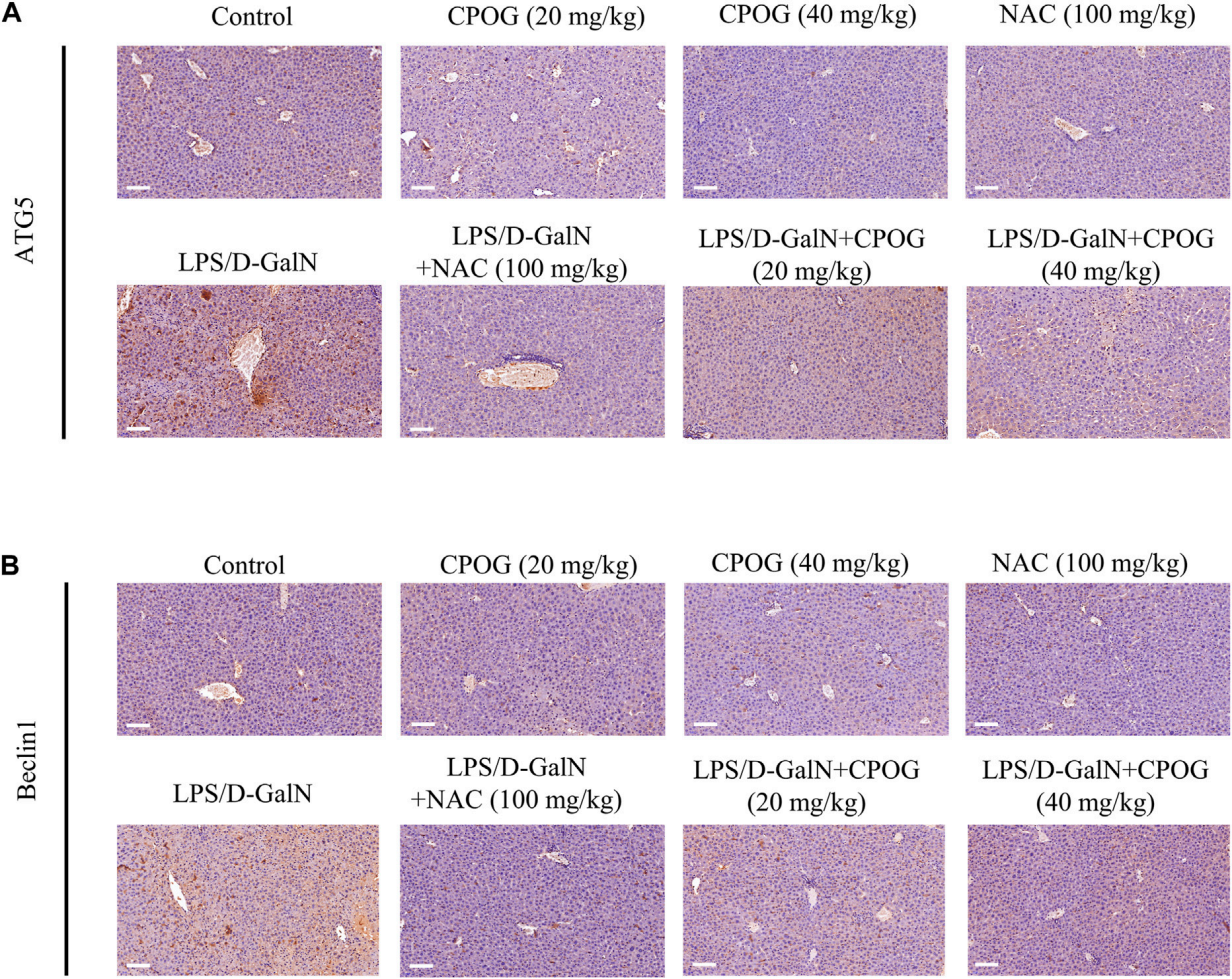
CPOG inhibited the expression of ATG5 and Beclin1 in LPS/D-GalN-induced ALF model. **(A,B)** Immunohistochemistry assay was performed with the indicated antibodies. The nucleus was stained with DAPI (blue color). Representative photographs were shown from vehicle mice, mice treated with CPOG (20 mg/kg), mice treated with CPOG (40 mg/kg), mice treated with NAC, mice treated with LPS/D-GalN, mice treated with LPS/D-GalN and NAC, mice treated with LPS/D-GalN and CPOG (20 mg/kg), mice treated with LPS/D-GalN and CPOG (40 mg/kg). Scale bar = 100 µm.

Then immunohistochemistry was performed to detect the expression of ATG5 and Beclin1 in liver tissue. As shown in Figures 4A,B, treatment with NAC or CPOG alone had no effect on the expression of ATG5 and Beclin1. LPS/D-GalN injection induced upregulation of ATG5 and Beclin1, which could be alleviated by the treatment with NAC or CPOG. All these results indicate that COPG alleviated LPS/D-GalN-induced autophagic response in ALF model.

### 3.4 CPOG alleviated LPS-induced oxidative stress and inflammation response in RAW264.7 cells

**FIGURE 5:**
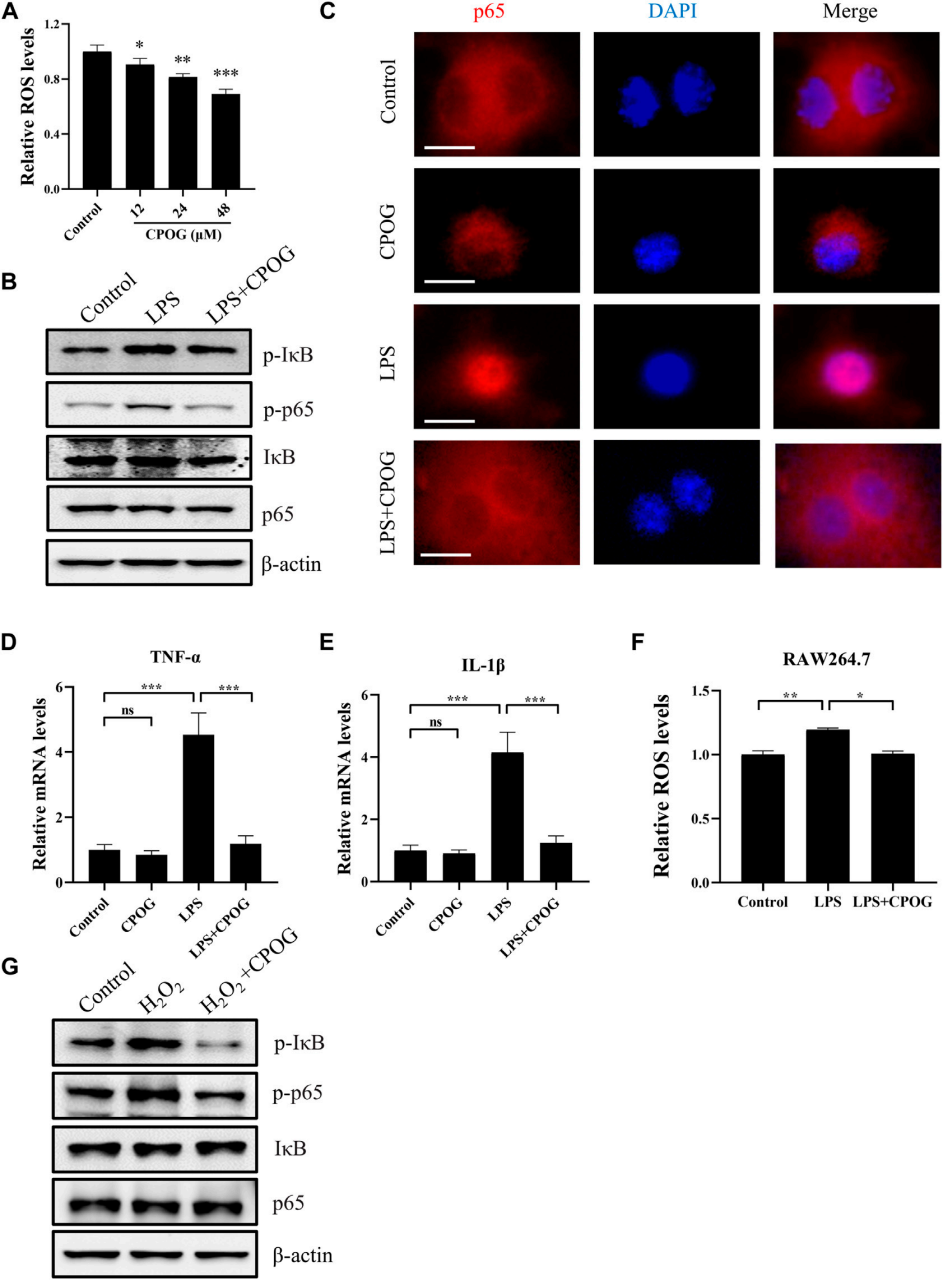
CPOG alleviated LPS-induced oxidative stress and inflammation response in RAW264.7 cells. **(A)** RAW264.7 cells were treated with indicated concentrations of CPOG and intracellular ROS level was detected with CM-H2DCFDA. **(B–F)** RAW264.7 cells were treated with 1 μg/ml LPS in the presence or absence of 48 μM CPOG for 4 h. The expression of indicated proteins was detected by Western blot **(B)**. p65 cell location was detected by immunofluorescence assay **(C)**. Expression of TNF-α **(D)** and IL-1β **(E)** was detected by RT-PCR. Intracellular ROS level was detected with CM-H2DCFDA **(F)**. **(G)** RAW264.7 cells were incubated with 48 μM CPOG for 4 h before treated with 500 μM H_2_O_2_ for 30 min. The expression of indicated proteins was detected by Western blot. Values represent mean ± S.D. Significance was determined by one-way ANOVA test. Data are representative of three independent experiments. *p < 0.05, **p < 0.01, ***p < 0.001.

CPOG treatment downregulated intracellular ROS level in dose-dependent manner without affecting cell viability in RAW264.7 cells (Figure 5A; Supplementary Figure S2A). In order to investigate the underlying mechanism of the anti-inflammatory effect of CPOG on LPS/D-GalN-induced ALF, we detected the expression of proteins in NF-κB signaling pathway in RAW264.7 cells. CPOG treatment alone had no effect on the expression of p-IκB and p-p65. The expression of p-IκB and p-p65 were upregulated after LPS treatment. By contrast, CPOG significantly inhibited LPS/D-GalN-induced phosphorylation of IκB and p65 (Figure 5B; Supplementary Figure S2B). Translocation of the NF-κB subunit p65 from cytoplasm to the nucleus is key to the activation of the inflammatory signaling pathway. Hence, the location of p65 was detected and the results showed that LPS-induced nuclear translocation of p65 was also reversed by CPOG treatment, which is in accordance with its phosphorylation change (Figure 5C). Next, we examined the effect of CPOG on the expression of IL-1β and TNF-α using q-PCR. LPS treatment increased mRNA levels of IL-1β and TNF-α, while the application CPOG effectively ameliorated the situation (Figures 5D,E).

LPS exposure may lead to increased reactive oxygen species generation, which is a potential activator of NF-κB signaling pathway. To determine if the anti-inflammatory effect of CPOG is related to its anti-oxidative property, ROS levels were detected following LPS treatment. Our results showed that LPS-induced oxidative stress was also alleviated by CPGO treatment (Figure 5F). Furthermore, H_2_O_2_ induced activation of NF-κB signaling was also blocked by CPOG (Figure 5G). In short, these results suggest that CPOG exhibited remarkably anti-inflammatory effect in association with its anti-oxidative property.

### 3.5 CPOG alleviated LPS-induced oxidative stress and autophagy in LX2 cells

**FIGURE 6:**
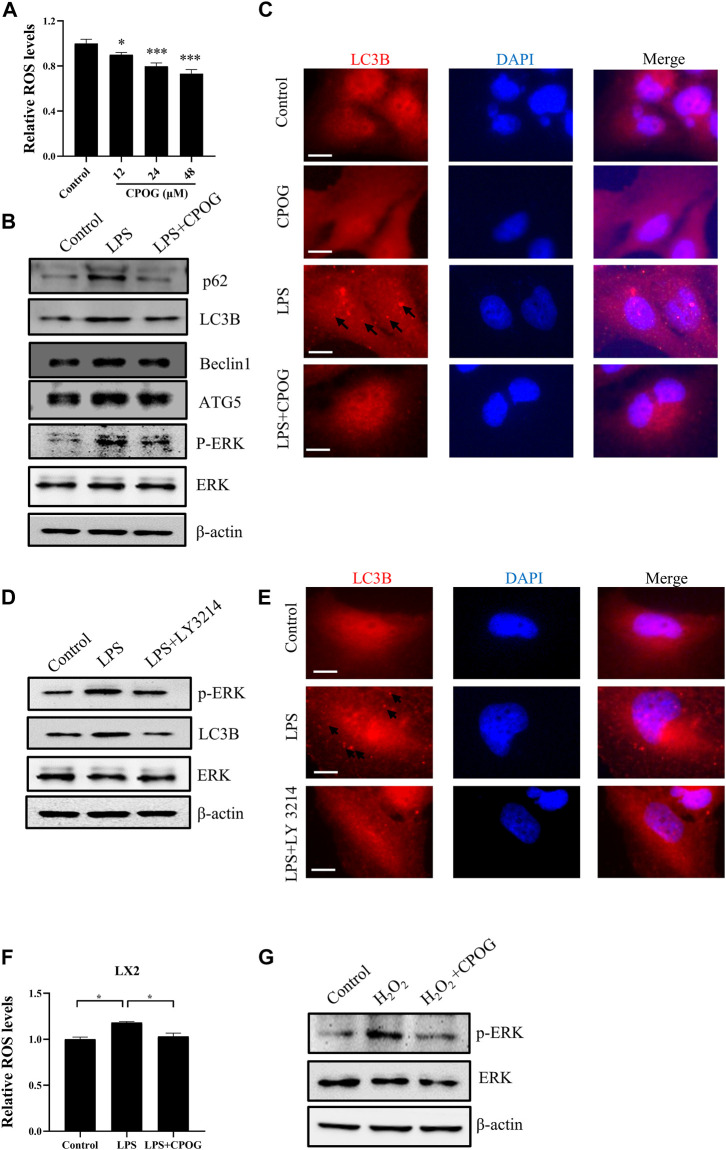
CPOG alleviated LPS-induced oxidative stress and autophagy in LX2 cells. **(A)** LX2 cells were treated with indicated concentrations of CPOG and intracellular ROS level was detected with CM-H2DCFDA. **(B,C)** LX2 cells were treated with 1 μg/ml LPS in the presence or absence of 48 μM CPOG for 4 h. The expression of indicated proteins was detected by Western blot **(B)**. Formation of LC3B puncta was detected by immunofluorescence assay **(C)**. Scale bar = 10 µm. **(D,E)** LX2 cells were incubated with LY3214 for 12 h before treated with 1 μg/ml LPS for 4 h. The expression of indicated proteins was detected by Western blot **(D)**. Formation of LC3B puncta was detected by immunofluorescence assay **(E)**. Scale bar = 10 µm. **(F)** LX2 cells were treated with 1 μg/ml LPS in the presence or absence of 48 μM CPOG for 4 h. Intracellular ROS level was detected with CM-H2DCFDA. **(G)** LX2 cells were incubated with 48 μM CPOG for 4 h before treated with 500 μM H_2_O_2_ for 30 min. The expression of indicated proteins was detected by Western blot. Values reWresent mean ± S.D. Significance was determined by one-way ANOVA test. Data are representative of three independent experiments. *p < 0.05, **p < 0.01, ***p < 0.001.

CPOG treatment downregulated intracellular ROS level in dose-dependent manner without affecting cell viability in RAW264.7 cells (Figure 6A; Supplementary Figure S3A). LPS mediated autophagic cell death induced by oxidative stress is associated with various disease. We tested the autophagy induction effect of LPS in liver cell lines. Our results showed the expression of LC3B and p62 is unaffected by LPS or CPOG treatment in LO2 cells (Supplementary Figure S3B). As shown in Figure 6B and Supplementary Figure S3C, the expression of LC3B, p62, ATG5, and Beclin1 in LX2 cells increased significantly when treated with LPS, which could be alleviated by the application of CPOG. MAPK signaling pathway played an important role in LPS-induced autophagy. Our results showed that ERK phosphorylation was upregulated by LPS, which was attenuated by CPOG treatment. Then, immunofluorescence assay was performed to detect the formation of LC3 puncta in LX2 cells. As shown in Figure 6C, LPS treatment induced the formation of LC3 puncta, which was alleviated by CPOG treatment. To confirm the role of ERK in LPS-induced autophagy, LX2 cells were treated with LY3214, an inhibitor of ERK, and the results showed that LPS-induced up-regulation of LC3B was alleviated by LY3214 treatment (Figure 6D; Supplementary Figure S3D). Immunofluorescence results showed that LY3214 treatment also alleviated LPS-induced formation LC3B puncta (Figure 6E). These observations indicate ERK activity was required for LPS-induced autophagy in LX2 cells. Previous studies indicated that ROS play a role as messengers to activate the mitogen-activated protein kinases (MAPKs) (Traore et al., 2008; Yuan et al., 2021). Therefore, the role of LPS-induced ROS in the activation of ERK was explored. Our results showed that LPS treatment increased ROS production in LX2 cells, which could be alleviated by CPOG (Figure 6F). Further, CPOG treatment alleviated H_2_O_2_-induced phosphorylation of ERK (Figure 6G). In conclusion, these results suggest that COPG alleviated LPS-induced autophagy by inhibiting ROS generation and ERK phosphorylation.

## 4 Discussion

Mice administrated with LPS/D-GalN is a well-established experimental model that resembles human ALF (Silverstein, 2004; Wilhelm et al., 2009). Cell-to-cell communication within the liver is a rising field to understand the liver pathogenesis, in which non-parenchymal cells may be directly targeted or activated in response to toxic pathogens (Robinson et al., 2016). In this study, we attempted to explore the therapeutic potential of CPOG in the treatment of ALF and its underlying mechanism. We demonstrated that CPOG could ameliorate liver damage induced by LPS/D-GalN and improved survival rates. Importantly, CPOG alleviated LPS/D-GalN-induced oxidative stress in liver tissue. LPS/D-GalN-induced release of AST and ALT as well as secretion of IL-1β and TNF-α was attenuated by CPOG *in vivo*. Moreover, CPOG alleviated LPS-induced ROS generation *in vitro* and caused significant reduction in IL-1β and TNF-α production in macrophages and inhibited autophagy as indicated by LC3 puncta formation in hepatic stellate cells.

ROS are by-products of metabolism of oxygen that includes non-radicals like H_2_O_2_ and ^1^O_2_ and free radicals like O^•−2^, OH^•^. Basal ROS production served as signaling molecule in cell survival and proliferation (Forman et al., 2010). However, excessive ROS production induced damage of nucleic acids, proteins, and lipids, and was involved in the pathogenesis of various diseases (Andersen, 2004; Trachootham et al., 2009). It was reported that LPS triggered ROS production by activating NADPH Oxidase four in macrophages and endothelial cells (Hsu and Wen, 2002; Simon and Fernandez, 2009). Here, it was found that LPS induced excessive ROS accumulation in liver tissue as well as in RAW264.7 and LX2 cells. CPOG treatment down-regulated intracellular ROS level and alleviated LPS-induced ROS production both *in vivo* and *in vitro*. These results suggest that CPOG is strong antioxidant that exerts beneficial effect against oxidative stress.

NF-κB is a family of transcriptional factors including p65, p52, p50, RelB, and c-Rel, which regulates genes involved in inflammatory and immune responses (Oeckinghaus and Ghosh, 2009). Notably, activation of p65 plays vital role in the release of pro-inflammatory mediators in macrophages in response to various stimuli (Giridharan and Srinivasan, 2018). LPS has been reported to induce expressions of many pro-inflammatory mediators like IL-1β and TNF-α through the activation of NF-κB signaling (Muniandy et al., 2018). Here, it was found that LPS induced up-regulation of IL-1β and TNF-α both *in vivo* and *in vitro*, which could be alleviated by CPOG treatment. IκB is an inhibitor of p65. Phosphorylation of IκB by multi-subunit IκB kinase facilitates its ubiquitin-dependent degradation, resulting in the phosphorylation and nuclear translocation of p65 (Li et al., 2019). Our results showed that CPOG inhibited the up-regulation of p-IκB and p-p65 induced by LPS. Translocation of p65 from cytoplasm to nucleus was also blocked by CPOG treatment. Our results suggested that CPOG inhibited LPS-induced inflammation response through NF-κB signaling pathway by suppressing IκB phosphorylation. Consistent with previous findings that oxidative stress is an activator of NF-κB signaling (Takada et al., 2003), we found that H_2_O_2_ treatment upregulated the expression of p-IκB and p-p65 in RAW 264.7 cells. CPOG treatment decreased intracellular ROS level and alleviated oxidative stress-induced activation of NF-κB signaling. These data suggest that the anti-inflammatory effect of CPOG was partly due to its antioxidant ability.

LPS-induced dysfunctional autophagy was reported to result in autophagic cell death through oxidative stress in various tissue (Xu et al., 2006; Lin et al., 2016; Liu et al., 2020). p62 and LC3 are two key factors in autophagosomes formation (Klionsky et al., 2010). In this study, we observed upregulated expression of LC3B, p62, ATG5, and Beclin1 followed by LPS treatment in mice liver tissue, which could be alleviated by CPOG treatment. In response to liver damage, hepatic stellate cells play a crucial role in liver fibrosis and scar tissue formation (Krizhanovsky et al., 2008). Our results showed that LPS treatment induced up-regulation of LC3B and P62 in LX2 cells but not in LO2 cells, indicating that LPS had distinct effect on different liver cell types and promoted autophagic activity in LX2 cells. The p38MAPK pathway is involved in a variety of physiological process such as cell proliferation, differentiation, and apoptosis (Zhang and Liu, 2002; Slobodnyuk et al., 2019). Raf/MEK/ERK activation is vital to the expression of LC3B and P62 (Kim et al., 2014). Intriguingly, expression of p-ERK also increased in LX2 cells following LPS stimulation, which was suppressed by CPOG treatment. In particular, pretreatment with ERK inhibitor (LY 3214) blocked LPS-induced LC3B expression and LC3B puncta formation in LX2 cells, suggesting that LPS-induced autophagy was mediated by MAPK signaling pathway. Moreover, it was reported that ERK could be activated in ROS-dependent manner (Deng et al., 2021). Here, it was found that H_2_O_2_ induced oxidative stress upregulated the expression of p-ERK. Application of CPOG significantly decreased ROS generation in LX2 cells and alleviated H_2_O_2_-induced ERK phosphorylation. These results indicate that CPOG alleviated LPS-induced autophagy in LX2 cells by inhibiting ROS-mediated ERK phosphorylation.

## 5 Conclusion

In conclusion, this study first demonstrated that CPOG protected mice from LPS/D-GalN-induced ALF. CPOG showed strong antioxidant ability both *in vivo* and *in vitro*. Molecularly, CPOG inhibited LPS-induced release of IL-1β and TNF-α by the inactivation of NF-κB signaling pathway in RAW264.7 cells. CPOG attenuated LPS-induced activation of MAPK signaling, therefore attenuated the expression of autophagy-related proteins and LC3 puncta formation in LX2 cells. Therefore, CPOG offers a potential therapeutic strategy for the cure of ALF, though further clinical trial is needed.

## Data Availability

The datasets presented in this study can be found in online repositories. The names of the repository/repositories and accession numbers can be found below: https://figshare.com/s/aa1575aafb9fd25145a2.

## Abbreviations

ALF, Acute liver failure; ALT, Alanine aminotransferase; AST, Aspartate transaminase; CPOG, Chrysophanol-8-O-glucoside; D-GalN, D-galactosamine; HSC, Hepatic stellate cell; LPS, Lipopolysaccharide; NPC, Non-parenchymal cell; ROS, Reactive oxygen species.
