# Supplementary material for: Chrysophanol-8-O-glucoside protects mice against acute liver injury by inhibiting autophagy in hepatic stellate cells and inflammatory response in liver-resident macrophages
Source: Front Pharmacol. 2022 Sep 6;13:951521. doi: 10.3389/fphar.2022.951521 (PMC9485814; doi:10.3389/fphar.2022.951521)
Supplement: Supplementary file 1 [file DataSheet1.pdf]

Supplementary Figure 1

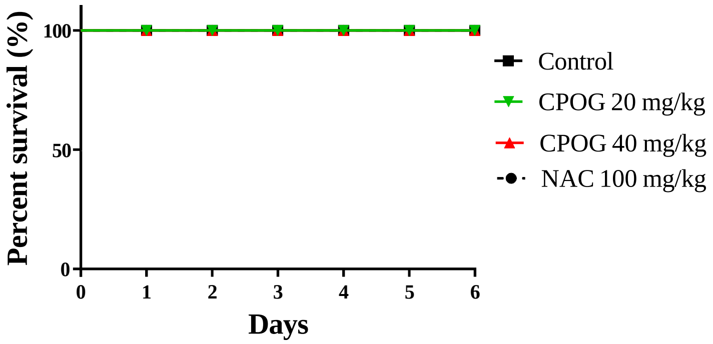

Supplementary Figure 1. Toxicity of NAC and CPOG in mice. Kaplan-Meier method was used to create the survival curves after CPOG or NAC administration.

Supplementary Figure 2

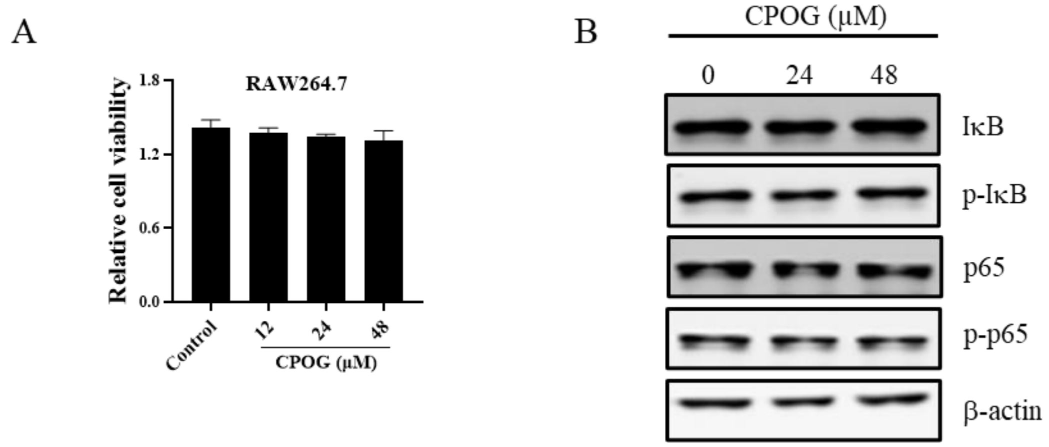

Supplementary Figure 2. Effect of CPOG on cell viability and NF-κB signaling in RAW264.7 cells. (A-B) RAW264.7 cells were treated with various concentrations of CPOG. Cell viability was analyzed by Cell Counting Kit-8 (A) and protein expression was detected by western blot (B).

Supplementary Figure 3

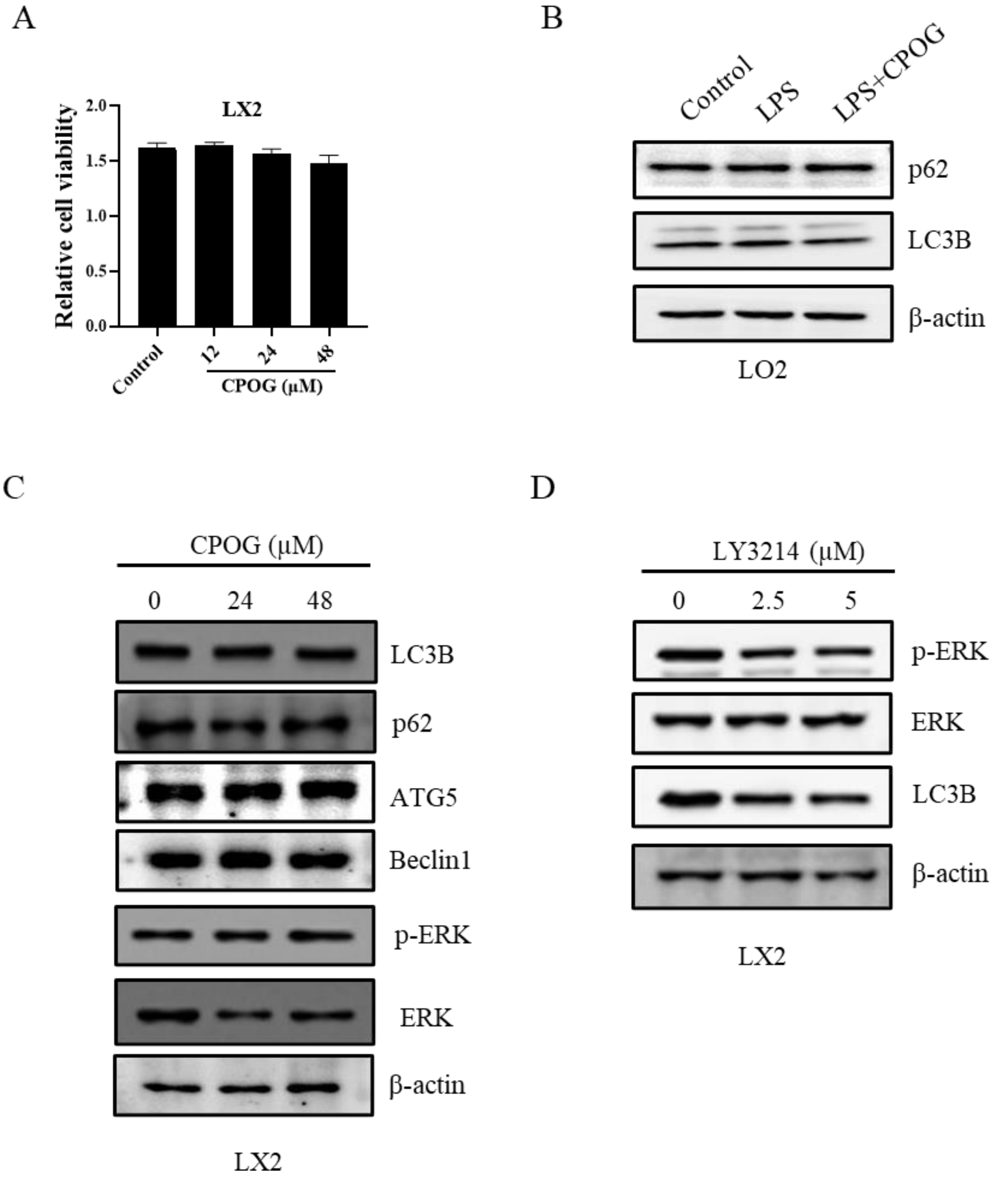

Supplementary Figure 3. Effect of CPOG and LY3214 on autophagy in hepatocytes. (A) LX2 cells were treated with various concentrations of CPOG. Cell viability was analyzed by Cell Counting Kit-8. (B) LO2 cells were treated with 1 μg/ml LPS with or without 48 μM CPOG for 4 h. The expression of proteins was detected by western blot with indicated antibodies. (C) LX2 cells were treated with various concentrations of CPOG. The expression of proteins was detected by western blot with indicated antibodies. (D) LX2 cells were treated with various concentrations of LY3214. The expression of proteins was detected by western blot with indicated antibodies.
